# First detected case of rabbit Haemorrhagic disease virus 2 (RHDV2) in the Irish hare (*Lepus timidus hibernicus*)

**DOI:** 10.1186/s13620-021-00205-2

**Published:** 2021-09-18

**Authors:** Aideen Kennedy, Louise Britton, Andrew W. Byrne, Christina Byrne, Mícheál Casey, Orla Flynn, Jose Maria Lozano, Ferdia Marnell, Maire McElroy, Neil Reid, Margaret Wilson, William FitzGerald

**Affiliations:** 1 Department of Agriculture, Food and the Marine (DAFM), Dublin, Ireland; 2 National Parks and Wildlife Service, Department of Housing, Local Government and Heritage, 90 King Street North, Dublin, Ireland; 3 Institute for Global Food Security (IGFS), School of Biological Sciences, Queen’s University Belfast (QUB), Belfast, UK

**Keywords:** RHDV2, Endemic species, Wildlife disease, Hare coursing, Calicivirus, RT-PCR

## Abstract

**Background:**

Rabbit haemorrhagic disease virus (RHDV) is a *Lagovirus*, a subgroup of the family *Caliciviridae*. RHDV2 is a variant first described in France in 2010, and has since spread globally. It has been reported in several Lagomorph species (rabbits, hares, and their relatives) as well as other mammals including voles and shrews. The disease has raised international concerns for its potential impact on population abundance trajectories, particularly as 25% of Lagomorphs are currently Red-Listed by the International Union for the Conservation of Nature (IUCN). The Irish hare (*Lepus timidus hibernicus*) is a subspecies of the mountain hare, *L. timidus*, and is endemic to Ireland, making it an Evolutionarily Significant Unit of intrinsic value.

**Case presentation:**

The first case of RHDV2 was detected in a wild Irish hare in July 2019. The individual exhibited atypical neurological behaviour (running in circles) prior to death. On necropsy, pink tinged foam was seen in the trachea and congestion was noted in the lungs, but there was no evidence of haemorrhages in any other organ. Both the liver and spleen were tested by reverse transcription real time qPCR confirming high levels of RHDV2 RNA. Histopathology confirmed multifocal necrotising hepatitis.

**Conclusion:**

The Irish hare is susceptible to RHDV2 infection. Further investigation is warranted to explore the clinical, epidemiological, and population biology implications.

## Background

Rabbit Haemorrhagic Disease (RHD) is caused by rabbit haemorrhagic disease virus (RHDV), a member of the genus *Lagovirus*, in the family *Caliciviridae* [1]. RHD was first described in China in the mid-1980s by Liu *et al.* [2] and the virus has since spread worldwide [1]. RHDV was used as a form of biocontrol for non-native invasive European rabbits (*Oryctolagus cuniculus*) in Australia and New Zealand where they are a pest causing considerable ecological damage [3]. Various forms of the disease have been reported ranging from peracute to chronic [1]. In peracute disease, rabbits exhibit no clinical signs and death is sudden. In acute forms, there is typically anorexia, apathy and there may be conjunctival congestion. Neurological signs have also been described including opisthotonus, excitability, paralysis and ataxia. In addition, respiratory signs can occur, such as foamy bloody nasal discharge [1]. High mortality has been reported, with rabbits typically dying within 48 h after the onset of fever [4]. In subacute forms, clinical signs are less severe and some rabbits will survive. Lastly, a chronic form with clinical signs of generalised jaundice, anorexia and lethargy occurs less frequently [1]. The primary tissues targeted by the virus include the liver, spleen and lung. Typical gross pathological findings are congestion and haemorrhage in the lungs, heart and kidneys due to disseminated intravascular coagulation [1]. Histopathological changes in the liver include periacinar necrosis, whilst individual hepatocytes undergo coagulation necrosis with strong hyper-eosinophilia and are often surrounded by karyorrhectic neutrophils [5].

In 2010, a new RHDV variant was identified affecting wild and farmed rabbits in France [6]. The virus, designated RHDV2, was considered a distinct serotype as it had a sufficiently different antigenic profile from that of the original RHDV. Additional distinguishing characteristics of RHDV2 infection include a slightly longer disease duration, more variable mortality rate ranging from 5–80% and the capacity to infect younger animals from 10–15 days of age [7]. RHDV2 also has the capacity to infect and cause disease in a wide range of lagomorph species including various hares (*Lepus* spp.) [8, 9]. RHDV2 has been reported throughout Europe in brown hares (*L. europaeus*), cape hares (*L. capensis*) [8], Italian hares (*L. granatensis*) [10] and mountain hares (*L. timidus*) [11]. It has also been identified in the North America cottontail (*Sylvilagus* spp.), Mexican volcano rabbit (*Romerolagus diazzi*), as well as non-lagomorphs including wood mice (*Apodemus* spp.), voles (*Microtus* spp.), shrews (*Crocidura* spp.) [12], and badgers (*Meles meles*) [13].

The Irish hare (*L. timidus hibernicus*) is one of 16 subspecies of mountain hare and represents the only lagomorph native to Ireland [14]; though non-native European rabbits and brown hare have been introduced. The Irish hare is of conservation interest [15] being listed as an internationally important species, designated under the EU Habitats Directive [16] and the Bern Convention [17]. It is legally protected in Ireland under the Wildlife Act (1976) and Wildlife (Amendment) Act 2000. The species declined dramatically during the twentieth century due to agricultural intensification. For example, an analysis of game bag records suggest declines of 88% during the twentieth century (1908–1970). Low densities are now typical at around *ca*. ~3hares/km^2^ and a total population of 223,000 individuals were estimated during winter 2018/2019 [17]. The following case report details the first report of RHDV2 in this species.

## Case presentation

### First case of RHDV2 in the Irish hare

A wild juvenile female Irish hare, which was observed running in circles prior to death, was submitted for necropsy on 25th July 2019 to the Kilkenny Regional Veterinary Laboratory, Department of Agriculture, Food and the Marine (DAFM) by a National Parks and Wildlife Service (NPWS) Conservation Ranger from the Wexford Wildfowl Reserve (52°21′32.1″N 6°25′11.8″W, Irish Grid Reference T 076239). The carcass measured 32 cm head-tail length and weighed ~800 g.

**Fig. 1:**
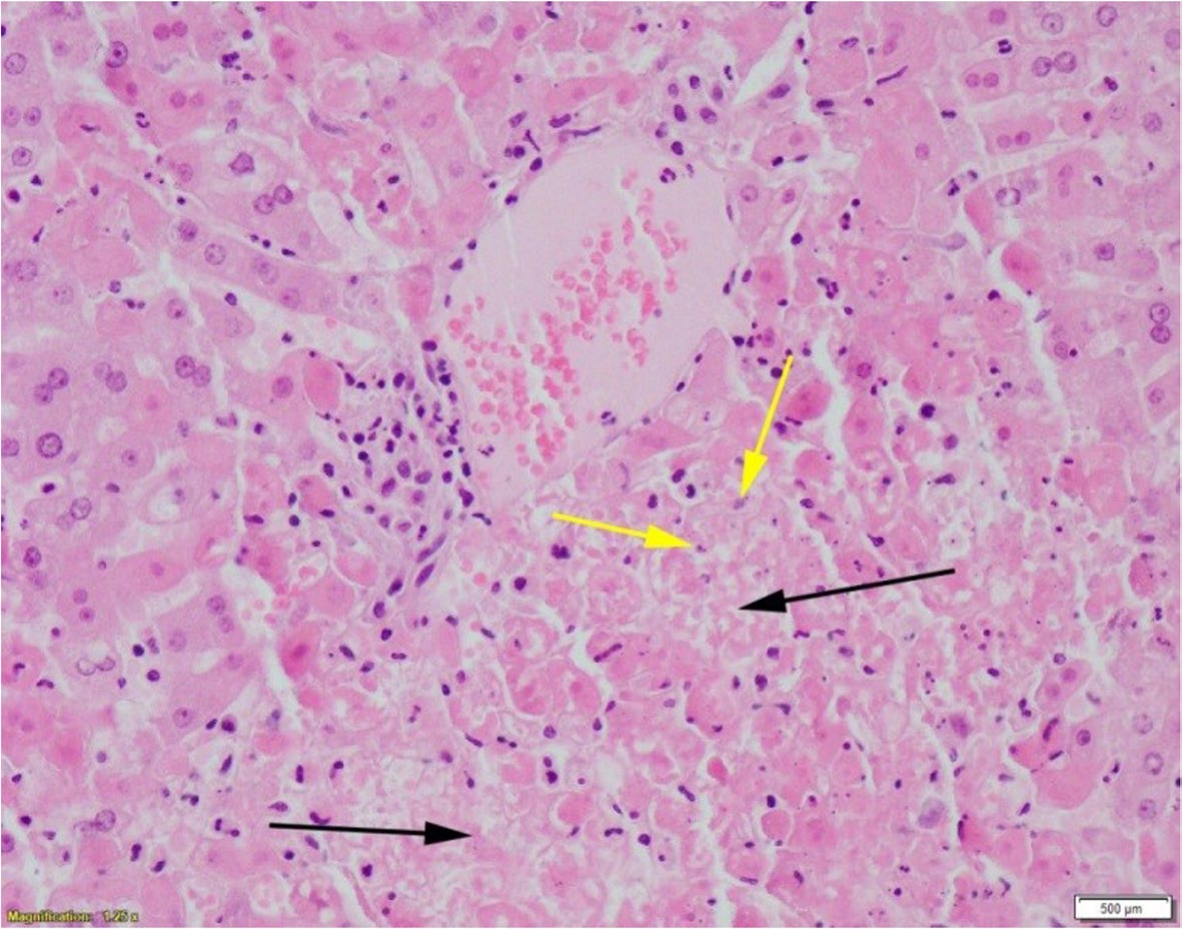
Photomicrograph of liver tissue illustrating severe acute periportal-submassive hepatic necrosis with cell lysis (black arrows) and nuclear karyolysis (yellow arrows). (Haematoxylin & Eosin, 10x, Margaret Wilson)

Gross findings included focal mild congestion in the lungs and a small volume of blood tinged froth in the trachea with a large volume of soft green faeces in the large intestine. No skin lesions or conjunctival congestion was noted. Lung and liver tissue were sampled for routine bacteriological culture. *Streptococcus gallolyticus*, a commensal of the gut and opportunistic pathogen of animals [18], was cultured from the lung. A faecal sample was taken for examination by the McMasters technique [19], detecting a strongyle count of 2500 eggs per gram. Sections of liver and spleen were fixed in neutral buffered formalin for histopathological examination. Histopathological examination of the liver showed severe acute multifocal random-submassive coagulative necrosis characterised by hepatocyte dissociation, swelling, hypereosinophilia and nuclear karyolysis and karyorhexis (Fig. 1). Multifocally, some necrotic areas were infiltrated by low numbers of heterophils and macrophages. Histopathological examination of the spleen showed mild depletion of the splenic red pulp.

Liver and spleen tissue was taken for detection of RHDV by reverse transcription real time polymerase chain reaction (RT-qPCR) [20]. Briefly, total RNA was extracted using the MagNA Pure 96 automated extraction system (Roche Diagnostics, Switzerland) according to the manufacturers protocol. RNA was eluted in 100 μl elution buffer. Reactions were set up in a PCR dedicated cabinet. RT-qPCR master mix was prepared using TaqMan Fast Virus 1-Step Master Mix (Thermofisher Scientific), primers RHDV2-forward and RHDV2-reverse (1 μM) and RHDV2-probe (0.15 μM). The primer and probe nucleotide sequences and positions in the vp60 gene are illustrated in Table 1. All reactions were made up to 20 μl with nuclease free water. Every run included a positive control (RHDV-2 WE 10213), kindly provided by Friedrich- Loefler-Institut, Insel Riems, and a no template control. The RT-qPCR was carried out using the AriaMx Real-Time PCR System (Agilent) in a 96 well optical plate format. Thermocycling conditions included one cycle at 50 °C for 45 mins for reverse transcription, followed by one cycle at 95 °C for 15 mins and 40 cycles of 95 °C for 15 s, 60 °C for 30 s and 72 °C 30 s. Fluorescence was acquired during the annealing step. Cycle to threshold values of 13 and 15 were generated from the liver and spleen samples, respectively, indicative of high viral loads.

**Table 1:** Nucleotide sequences and position within vp60 gene for RHDV2 RT-qPCR primers and probe

Oligonucleotide	Sequence (5′-3′)	Position in vp60 gene
RHDV2-F	TGGAACTTGGCTTGAGTGTTGA	1571–1592
RHDV2-R	ACAAGCGTGCTTGTGGACGG	1678–1697
RGDV2 probe	FAM-TGTCAGAACTTGTTGACATCCGCCC-BHQ1	1664–1640

In summary, histopathological findings, in combination with molecular results, were consistent with a diagnosis of RHDV2 infection.

## Discussion and conclusion

This is the first case report of RHDV2 in the Irish hare (*Lepus timidus hibernicus*) – an endemic subspecies of conservation concern only found on the island of Ireland. Although RHDV2 has been reported in domestic rabbits since 2016, this is the first published report of RHDV2 in a wild host in Ireland [21]. It is currently unknown how the pathogen arrived to Ireland, however several routes could be speculated about including the virus entering the country on a fomite, like on clothes or boots of traveller(s). The identification of RHDV2 circulating in wild populations has been associated with declines in lagomorph abundance in several countries [22, 23]. In Australia, where non-native European rabbits were controlled using released strains of RHDV, the emergence of RHDV2 has lead to up to 60% decline in abundance at monitored sites [24, 25]. Notable declines of rabbits have been reported in Spain and Portugal [22, 23]; the latter also associated with declines in other predators that prey primarily on rabbits [23]. Outbreaks have been reported in several subspecies of hares in Europe (e.g. [8, 11, 26]), with evidence of spillover of infection from rabbits to wild hare populations. Evidence suggests that the pathogen may be virulent to several of these species including mountain hares *L. timidus* [11, 26, 27]. Hare-to-hare transmission of RHDV2 appears to be possible, with the maintenance of infection in an island population of Mountain Hares (geographically isolated from rabbits) in Sweden for a number of months being reported by Niemenis et al. [11]; in that case, infection may have been maintained within the population either by environmental contamination (indirect transmission) or by multiple incursions of infection into the population from the mainland (where there was a rabbit RHDV2 outbreak). It is currently unknown how RHDV2 was first transmitted to wild hares in Ireland, but it is notable that the first cases were found in domestic rabbits, and one could speculate that a spillover event(s) may have seeded infection into wild populations of rabbits and hares. Furthermore, it is also unknown the impact of RHDV2 on Irish hare population abundances, or their predators (such as Irish stoats *Mustela erminea hibernica*, buzzards *Buteo buteo* or foxes *Vulpes vulpes*).

As the Irish hare is a unique subspecies found only in Ireland, possible impacts of the virus on the national population is concerning, particularly with relevance to the practice of hare coursing. In Ireland, hare coursing, the pursuit of a live hare by two competing muzzled greyhounds, remains legal under the Wildlife (Wild Mammals) (Open Seasons) Order 2005. Participation is widespread, with up to 6000 hares captured from the wild each year under Government licence. This process involves some animal handling and animals being held in enclosed “parks” for up to eight weeks prior to an event [28]. Surviving animals are returned to the wild [28], primarily at the location of capture, facilitated by ear tagging each hare. Following the finding of RHDV2 in the Irish hare, the Irish Coursing Club (ICC) license was temporarily revoked before limited capture of hares was permitted to resume, in conjunction with official monitoring and surveillance of the disease by the NPWS and DAFM. During this monitoring programme in 2020, approximately 400 hares were caught at a number of disparate locations in Ireland as part of this surveillance program. These animals were sampled and tested, with no evidence of circulating virus within this captured cohort during this period.

Given the conservation and cultural importance of the Irish hare in Ireland, further research is necessary to establish the distribution, prevalence, epidemiology, and population biology impact of RHDV2 on the Irish hare.

## Data Availability

All data are presented within the paper.
